# Depleting stabilized GOF mutant p53 proteins by inhibiting molecular folding chaperones: a new promise in cancer therapy

**DOI:** 10.1038/cdd.2016.145

**Published:** 2016-12-09

**Authors:** Evguenia M Alexandrova, Ute M Moll

**Affiliations:** 1 Department of Pathology, Stony Brook University, Stony Brook, NY, USA

*TP53* is the most frequently mutated gene in human cancer. One unusual feature that adds to the exceptional status of p53 is that the vast majority (75%) of p53 alterations are missense mutations in the DNA-binding domain.^1^ They fall into ‘DNA-contact' and ‘structural/conformational' mutant classes, which roughly – but not in every case – correlate with the degree of biophysical protein instability. Most if not all of these substitutions generate conformationally aberrant full-length mutant p53 (mutp53) proteins that have lost tumor suppressor functions and can have dominant-negative activities via hetero-oligomerization with wild-type p53. Importantly, however, many p53 mutants also acquire oncogenic gain-of-function (GOF) activities that actively promote malignant progression, cancer metabolism, stemness, invasion, metastasis and chemoresistance.^2, 3^

mutp53 GOF is strongly supported by knockin mouse models and the human data.^2, 4^ For example, R248Q mutp53 knockin mice have significantly earlier tumor onset and shorter survival than p53-null mice.^4^ Moreover, germline Li-Fraumeni syndrome (LFS) patients carrying R248Q missense mutations have significantly earlier cancer onset (by 10.5 years) and higher tumor burden than LFS patients with *TP53* loss-of-function (LOF) mutations.^4^ Similarly, sporadic human cancers across six major tumor entities show higher mortality (2-fold higher hazard ratios) for GOF mutant R282 and R248 patients, compared with p53 LOF mutant patients.^5^

mutp53 proteins undergo massive constitutive stabilization specifically in tumors (but not in normal tissues of knockin mice), and this stabilization is prerequisite for GOF.^2^ Currently, about 11 million patients worldwide live with cancers expressing highly stabilized mutp53. This raises the question: Is mutp53 itself an important therapeutic target? Compelling genetic evidence suggests so. Thus, RNAi-mediated mutp53 depletion has strong cytotoxic effects in human cancer cell lines *in vitro* and in xenografts.^3^ In allografts, knockdown of mutp53 in Kras^G12D^ pancreatic cancer cells strongly reduces their metastatic ability.^6^ Finally, in a conditional inactivatable (‘floxable') autochthonous mouse model expressing R248Q mutp53, we showed that allele ablation extends animal survival by 37%, induces regression or stagnation of advanced tumors and strongly suppresses metastasis.^7^ These data indicate that stabilized mutp53 is a tumor-specific vulnerability that can be pharmacologically exploited. Thus, understanding the mechanisms of mutp53 stabilization will be key for translation into therapy.

The heat shock protein HSP90 chaperone machinery is highly activated in cancer versus normal tissues, rendering them resistant to proteotoxic stress by supporting proper folding – and preventing aggregation – of conformationally aberrant oncoproteins including mutp53.^8, 9^ Both classes of mutp53 (structural and DNA-contact) require HSP90 for protection from degradation by their E3 ubiquitin ligases Mdm2 and CHIP (Figure 1a). Thus, combined inhibition of Hsp90 and its obligatory regulator cytosolic HDAC6 by small molecule inhibitors 17DMAG and Vorinostat/SAHA, respectively, or by Hsp90 inhibitor ganetespib alone extends overall survival of R175H (structural class) and R248Q (DNA-contact class) mutp53 animals by 30–59% and strongly prevents T-lymphomagenesis (the main tumor type in these mice). Surprisingly, although these are pleiotropic drugs, in no instance do p53-null mice benefit from Hsp90 inhibition^7^ (Figure 1a). These anti-cancer effects are concomitant with mutp53 degradation and cancer cell apoptosis, indicating tumor addiction to highly stabilized mutp53. Of note, a positive feed-forward loop from mutp53 to Hsp90 may further reinforce mutp53 stabilization (Figure 1a, green arrow). Thus, in HER2/EGFR-positive breast cancer, GOF mutp53 R175H activates the master heat shock transcription factor Hsf1, which in turn upregulates the heat shock response including HSP90, which then further stabilizes mutp53, HER2 and EGFR.^9^ This loop likely contributes to the vulnerability of such cancers to Hsp90 inhibition. Hsp90 inhibitors are currently in clinical evaluation for cancer and other diseases, although none has reached FDA approval.

In *Nature Cell Biology*, Parrales *et al.*^10^ now identified DNAJA1, an Hsp40 isoform, as another important molecular chaperone promoting mutp53 stabilization in cancer. In a drug screen for factors that degrade mutp53 without affecting wild-type p53, they found that statins, cholesterol-lowering drugs, degrade structural (but not DNA-contact) class mutp53 proteins. Moreover, statins specifically suppress growth of structural mutp53 cancer cells *in vitro* and in xenografts (cytostatic). Mechanistically, statins work via the mevalonate pathway/Hsp40/CHIP axis (Figure 1b). Reduction of mevalonate-5-phosphate (MVP) via inhibiting HMG-CoA reductase by statins – somehow – liberates structural mutp53 from the protective interaction with DNAJA1 and induces CHIP-mediated nuclear export, ubiquitination and degradation of mutp53. These data provide strong evidence for a second tangible therapeutic anti-mutp53 strategy besides Hsp90 inhibition. It would be interesting to test Hsp90i and statins in combination. Also, the effect of statins should be confirmed in knockin mouse models with native tumor stroma and uncompromised immune system.

Both Hsp40 and CHIP interact with Hsp70, and Hsp70 cooperates with Hsp90 in stabilizing mutp53.^11^ Yet, Hsp70 was not implicated in mutp53 degradation by statins, although it should be noted that only the constitutively expressed Hsc70, but not the stress-induced cancer-relevant Hsp70, was analyzed.^10^ The Hsp40/DNAJ family of co-chaperones modulate the activity of Hsp70 chaperones by stimulating their basal ATPase activity and substrate affinity,^8^ whereas CHIP (carboxy-terminus of Hsp70-interacting protein) promotes degradation of Hsp70-bound misfolded proteins. Hsp70 and Hsp90 are found in the same cancer-associated ‘epichaperome' complex.^8, 12^ Thus it is possible that other heat shock proteins besides Hsp90 and DNAJA1 protect mutp53 from statins-induced degradation.

Statins are in wide clinical use as cholesterol-lowering drugs, but in epidemiological studies have long been suspected to have cancer chemoprevention ability.^13^ Although this notion remains controversial, the findings of Parrales *et al.* might provide a possible mechanism. To resolve this important issue in future studies, the patients' mutp53 status, including structural versus DNA-contact class should be taken into account. One limitation is that the conformations of many ‘non-hot-spot' mutp53 proteins are unknown. Interestingly, the majority of published studies analyzed patients who at the time of their cancer diagnosis had already been on statins, possibly suggesting that statins might exert anti-cancer effects in clinically advanced tumors.

Intriguingly, upregulation of the mevalonate pathway is one of mutp53's GOF. mutp53 upregulates expression of many mevalonate pathway genes by binding to and promoting the activity of the sterol biosynthesis master transcription factor SREBP^14^ (Figure 1b, green arrow). Moreover, not just mutp53, but also mevalonate pathway upregulation correlates with poorer prognosis in breast cancer patients.^14^ Thus, by stimulating the mevalonate pathway, mutp53 further reinforces its own DNAJA1/MVP-mediated stability, which could further sensitize mutp53 to statins.

In sum, there are now two heat shock pathways mediating GOF mutp53 stabilization. Importantly, both are druggable and produce anti-tumoral effects. This further boosts the notion that indirect chaperone interception to degrade stabilized mutp53 is a more tangible strategy than the elusive long-term goal of identifying small molecules able to convert the many different aberrant conformations of mutp53 into a functional wild-type-like protein.

## Figures and Tables

**Figure 1:**
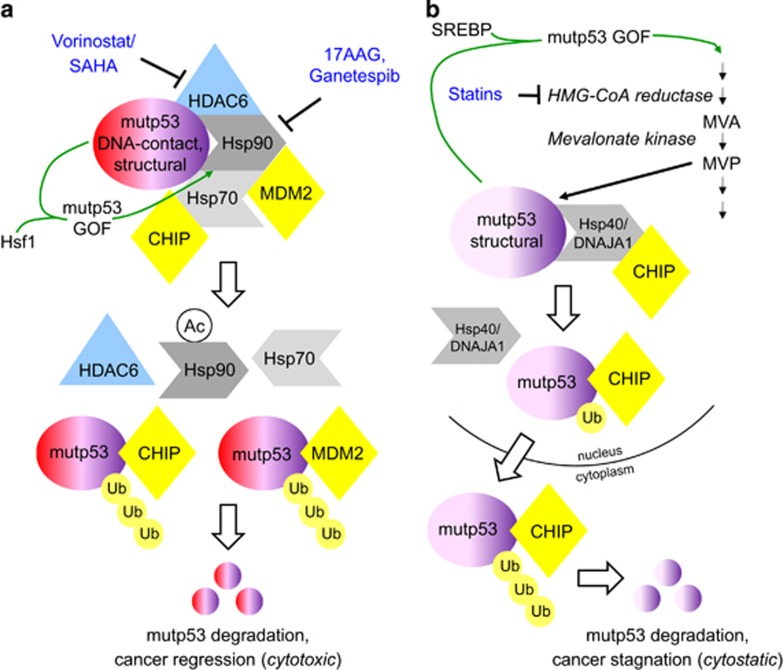
Degrading aberrantly stabilized mutant p53 proteins by drugging molecular heat shock folding chaperones. (**a**) The HSP90 chaperone machinery, including Hsp70, Hsp90 and its co-factor HDAC6, protects both classes of missense mutant p53 (DNA-contact and structural/conformational) from ubiquitination and degradation by the E3 ubiquitin ligases Mdm2 and CHIP. HDAC6 inhibitor Vorinostat (aka SAHA) and Hsp90 inhibitors 17AAG/17DMAG and Ganetespib promote mutp53 poly-ubiquitination and degradation, leading to apoptosis and regression of xenografts, allografts and autochthonous tumors (cytotoxic effect). Conversely, mutp53 upregulates the HSP90 machinery (as well as other stress chaperones, including Hsp40) by activating Hsf1, the master transcriptional regulator of the entire inducible heat shock response (green arrow). This represents a mutp53 GOF and a positive feedback loop for mutp53 stabilization. (**b**) Hsp40/DNAJA1 protects the conformational, but not the DNA-contact, class of mutp53 proteins from CHIP-mediated degradation. Statins inhibit HMG-CoA reductase in the mevalonate pathway, thus reducing levels of mevalonate-5-phosphate (MVP) that is normally required for protective mutp53–Hsp40 interaction, leading to CHIP-mediated nuclear export, poly-ubiquitination and degradation of this class of mutp53. This results in a cytostatic effect on tumor cells. Conversely, mutp53 can stimulate the mevalonate pathway by binding to and activating the sterol biosynthesis master transcription factor SREBP (green arrow), which is another mutp53 GOF and positive feedback loop for mutp53 stabilization. Overall, the two parallel pathways show symmetric architecture
